# The place of solar power: an economic analysis of concentrated and distributed solar power

**DOI:** 10.1186/1752-153X-6-S1-S6

**Published:** 2012-04-23

**Authors:** Vanessa Arellano Banoni, Aldo Arnone, Maria Fondeur, Annabel Hodge, J Patrick Offner, Jordan K Phillips

**Affiliations:** 1 The College, The University of Chicago, 5801 South Ellis Ave., Chicago, IL, USA

## Abstract

**Background:**

This paper examines the cost and benefits, both financial and environmental, of two leading forms of solar power generation, grid-tied photovoltaic cells and Dish Stirling Systems, using conventional carbon-based fuel as a benchmark.

**Methods:**

First we define how these solar technologies will be implemented and why. Then we delineate a model city and its characteristics, which will be used to test the two methods of solar-powered electric distribution. Then we set the constraining assumptions for each technology, which serve as parameters for our calculations. Finally, we calculate the present value of the total cost of conventional energy needed to power our model city and use this as a benchmark when analyzing both solar models’ benefits and costs.

**Results:**

The preeminent form of distributed electricity generation, grid-tied photovoltaic cells under net-metering, allow individual homeowners a degree of electric self-sufficiency while often turning a profit. However, substantial subsidies are required to make the investment sensible. Meanwhile, large dish Stirling engine installations have a significantly higher potential rate of return, but face a number of pragmatic limitations.

**Conclusions:**

This paper concludes that both technologies are a sensible investment for consumers, but given that the dish Stirling consumer receives 6.37 dollars per watt while the home photovoltaic system consumer receives between 0.9 and 1.70 dollars per watt, the former appears to be a superior option. Despite the large investment, this paper deduces that it is far more feasible to get few strong investors to develop a solar farm of this magnitude, than to get 150,000 households to install photovoltaic arrays in their roofs. Potential implications of the solar farm construction include an environmental impact given the size of land require for this endeavour. However, the positive aspects, which include a large CO2 emission reduction aggregated over the lifespan of the farm, outweigh any minor concerns or potential externalities.

## Background

Carbon-based fuel sources are becoming a hot commodity as the domestic electric industry watches the future. Proponents of renewable energy argue that an alternative approach will allow for more sustainable energy usage, help support future growth, avoid price spikes, allow for energy independence, and ultimately help slow the progression of global warming. To help illustrate such approach, consider a solar farm composed of Stirling engines covering an area of 100 squared miles. This alone could replace all the coal burned to generate energy in the United States [1].

Despite these positive externalities, the potential of major cost inequality and the associated fixed costs of renewable resources fuels debates. Renewable energy must combat the already present, tested, cheap, and ultimately reliable methods currently used to generate power.

This paper examines the cost and benefits, both financial and environmental, of two leading forms of solar power generation, grid-tied photovoltaic cells (PVs) and Dish Stirling Systems (DSS), using conventional carbon-based fuel as a benchmark. First, it will establish the manner in which these technologies, PVs and DSS, will be implemented in our study. Secondly, it will define a model city, its location, characteristics and constraints, which will be used as a parameter to evaluate the benefits and costs of each technology. Finally, it will attempt to determine whether decentralized photovoltaic farming is more effective and sustainable than a central, Stirling-engine based solar farm for our model city, with calculations related to fixed costs (construction, core technology used, land) and variable costs (labor, upkeep) determining the final prices of each power source. Our ultimate conclusion will be based on which power source is better from a consumer standpoint.

## Methods

### Setting concentrated solar power and distributed solar power exemplifiers

As the still immature solar energy market has grown we have learned more about different technologies and their ideal application. On the one hand, the flat panel photovoltaic cells, typically made of silicon, are the best-known form of solar technology [2], while the Concentrated Solar Power (CSP) industry is still at its infancy. While both provide a means of electricity production, this study is concerned with finding out which is the optimal means of energy consumption for a standard, West Coast suburban area.

When designing the large scale, high-priced solar farms, CSP is much preferred due to its cost effectiveness. However, CSP requires a large amount of room and very large-scale equipment to be most effective. Additionally, most recent plant installations have shown that economies of scale are applicable and therefore, as plant size increases, capital costs decrease [3]. Given this information we have chosen one of the most promising technologies, the Dish Stirling system, as our large-scale electricity producer.

Comparatively, photovoltaic energy production is far more effective when used in a decentralized manner due to its intrinsic properties, like its smaller size, which allows for more flexibility in the size of an installation. The household installable PV cells allows for single home power generation, with a surplus sent back to the grid for profit. These cells, though expensive, are often accompanied by a tax incentive. This allows for an analysis of decentralized means of power production without the large scale fixed costs of a central producer.

### Creation of a model city

Our goal is to get an accurate representation of the power needs and consumer habits of a typical city. In order to better account for variances and external influences, such as city demographics and weather, we decided to create a model city to test the two methods of solar-powered electric distribution.

### Model city location and the potential of the sun

The United States is of considerable interest for this study as it receives an enormous amount of solar heat when compared to the rest of the world. Each year the Earth intercepts a large amount of radiant heat, equaling roughly 5 x 10^20^ kilocalories. Thought of in terms of area, a typical square foot of land in the United States receives more than 1 kilocalorie per square foot, per minute or 500 kilocalories per day. Aggregated over an acre, those 40,000 square feet receive 20,000,000 kilocalories per day. Now, a conservative estimate for energy usage derived from coal, barrels of oil, and cubic feet of gas is somewhere around 150,000 kilocalories per day. When compared with the above stated estimate for light energy, the Sun could supply 2,000 times the heat energy currently used in the United States. Though promising, the illusive issue still remains, turning the potential energy into useful, usable electric energy.

It becomes very obvious that location is of prime importance for successful solar farming and energy production. The intensity of solar radiation outside of the Earth’s atmosphere is about 1,300 watts per square meter. We must assume that some of this is lost in the haze and cloud cover, leading to an estimate of 80-90% of the solar radiation successfully entering the atmosphere and reaching the ground. For simplicity we estimate this amount to be 1.100 kilowatts per square meter. The composition of light that enters is also of great importance, as it determines the applicable technology. The rays of sunlight are composed of diffuse light (scattered) and also direct rays from the sun (normal radiation). The above factors of haze, humidity and cloud cover can affect the light distribution and lead to increased scattering. As described by Leitner, flat panel PV power plants use both diffuse and direct radiation, while CSP can only harness the direct sunlight.

California is a prime location due to its latitude, low cloud cover and humidity, and the amount of sunlight received, as well as its great government incentives. This also works for PV, but further modifiers are required for the proposed solar farm. The large land requirements are not difficult to find, especially in the Western deserts of the United States. Not only is space plentiful, but also the conditions are ideal. This land required must be flat, as well as corresponding with other potential limiting factors. These factors, which affect the size of the land available, include military bases, national parks and protected wilderness, cropland, and developing urbanization. According to Leitner, land can be categorized into three resource classes of average solar energy resource (kWh/m2/day): 6.0 to 6.5 (good), 6.5 to 7.0 (great), and 7.0 and above (excellent). Given these factors, careful analysis reveals the Mojave Desert as an optimal location, despite its dwindling size, due to its flatness, availability of sun, and its proximity to major load centers.

Given this location, this study assumes all PV arrays will be facing between southwest and southeast at an elevation of around 30° as this maximizes solar energy production. Shading should also be taken into account, bearing in mind the proximity of local buildings, vegetation and the possible future plans of development or tree growth. Even minor shading can have a significant effect because it is the cell of lowest illumination that determines the current. This is why we set the following characteristics for our model city.

### Characteristics of model city

Using data from the Census Bureau we estimated that an average American city is composed of 150,000 households. Though more narrowed, city is still a wide term – often composed of mixed residential and commercial space. To further simplify things we decided that our city would be composed solely of residences, much like a suburb close to a metropolitan area. This allowed us to focus our findings on residential consumers, eliminating commercial and industrial electricity use. Furthermore, our model city does not include apartment high rises or town homes.

As for the residences themselves, the average American home is 2349 ft^2^ in area [4] and an average Californian residence consumes approximately 6960 kilowatt hours of electricity per year. Following a discussion with Executive Planner, Jim Christensen from Pacificorp we found out that to power a city of this size, 353,350,000ft^2^, we would need to generate 120 megawatts of power. In the case of our solar farm, we have to take into account the 7% average loss through the transmission lines. For the sake of conservatism and round numbers, we rounded this 8.4 megawatt loss up to 10 megawatts bringing the total to 130 megawatts.

We will take this model city and utilize it in each of our two case studies. First, we will analyze the requirements of meeting this hypothetical city’s needs entirely with residential photovoltaic arrays, with each household equipped with an array of solar panels necessary to meet the household’s own electrical needs. For our concentrated dish Stirling engine farm, power will be transmitted from a remote location to the model city. This second case requires the construction of power substation to lower the high voltage being transmitted from the farm into a safer level that can be utilized in homes. The costs of this added piece of capital, along with all the power source-based calculations, will be detailed in the Results & discussion section under the subtitle, “The case for concentrated Dish Stirling generation”.

### Constraining assumptions of photovoltaic technology

Given the geographical location of our model city, this study will assume that the array receives five equivalent noontime hours of sun exposure on an average day. This is a slightly conservative estimate; the state’s two largest metropolitan areas, Los Angeles and the San Francisco Bay, receive 5.6 and 5.4 noontime hours of sun on the average day, respectively, while other parts of the state receive as much as 7.7 average equivalent noontime hours per day [5]. For simplicity’s sake, this analysis also assumes an array generates no electricity outside of noontime hours.

Given that most photovoltaic cells are guaranteed to remain at 80% of starting efficiency after 25 years, as referenced by Black, this analysis will assume that the cells lose generating capacity at a compounded .9% per year. Thus, it will also limit its lifespan to the first 25 years and assume the array possesses no generating capacity afterwards.

Another assumption is the number of times the inverter has to be changed. Over time, the inverter coils wear down and eventually fail. Though there is not yet a consensus over the average life of a photovoltaic array’s inverter, estimates range from as little as 4.7 years [6] to longer than the lifespan of the array. For the sake of this analysis, we assume one inverter replacement half way through the lifespan of the array.

Finally this study will only take into account governmental policies that affect the whole state. Particularly, it will consider the 30% Resident Renewable Energy Tax Credit offered by the federal government, and the subsidies offered by the California Solar Initiative. However while several cities and counties offer additional incentives for photovoltaic array installations, these will be ignored for the purposes of this paper [7]. Similarly, this paper will not assume tiered electricity pricing as it is only active in certain parts of California, but it will mention how this may affect our findings.

Research shows that the average efficiency of these cells lies between 13% and 16% [8]. This loss in energy results from thermodynamic efficiency losses (up to 75%), losses in the inverter (10-15%), reflectance losses (~10%), temperature and dust accumulation (10%), and resistive electrical losses (1-3%) [9]. Hence, for the sake of conservatism this study will assume a 13% of cell efficiency. See Additional File 1 for further explanation on how energy is lost and further detail on how this technology works.

### Constraining assumptions of concentrated solar plant

Given the general location of California this study will set its hypothetical solar plant, for precision’s sake, outside the city of Barstow, in the county of San Bernardino. Hence, the plant will be affected by its typical weather of 102°F in the summer, receiving 281 days of sun, and 22 days of precipitation, with annual rainfall of 5 inches.

To determine the value of the land per acre we did the following research. In a ground known as the Mojave Desert Land Trust land prices ranges from $500 [10] to $1,522 [11] per acre depending on the government subsidy. Outside the realm of nature preservation the land prices begin to increase steadily. A survey of available land in Barstow reveals prices of $900 per acre in more rural areas [12] compared to $2,163 [13] and $4,225 [14] per acre closer to the city center of Barstow. Given the requirements of our project we took the average of the three that best meet our land qualities: $500, $900, and $1,522, establishing a cost of $974 per acre.

When it came to defining the lifespan of the plant we found many studies citing a theoretical lifespan ranging from 20 to 30 years. Sean Gallagher, Vice President of Market Strategy & Regulatory Affairs at Tessera Solar, provided a way to think of things more concretely for the sake of our study: the lifetime of a dish Stirling engine is 100,000 hours of run time. Now, given that our dishes will run 12 hours a day we get 100,000/12 = 8,333.33 days of lifetime or 22.83 years. For simplicity’s sake and the potential of downtime due to maintenance in the lifetime of the dishes, we set a lifespan of 23 years.

Similarly, over the lifetime of the farm certain routine maintenance would have to be performed. These include a complete washing of the reflective mirrors of each engine eight times a year, as well as engine maintenance once every two years. However, for calculations’ sake Sean Gallagher provided another way of determining the costs by calculating maintenance on a kilowatts per hour basis. This is done by defining the amount of grid-ready kilowatt-hours a dish generates in a year and by establishing a cost per kilowatt-hour of electricity generated. This logic shows that the cost of maintenance per kilowatt-hour of electricity generated is less than 2 cents, our case study assumes a cost of 1.8¢ per kilowatt-hour.

Next we define the sale price of energy produced with this technology. Several studies, including Black and Goodward, have quoted a sale price between 6¢ and 8¢ per kilowatt-hour [15], and given that this conservative range is outpaced during peak demand where many areas of California reach 11.33¢ per kilowatt-hour, this study will set the sale price at 8¢.

Finally, we set the initial rate of return (IRR). Given that there are no major doubts related to this technology as it has been tested and proven reliable, but also given that this is quite a large installation and certain speculation remains, as sustained by Leitner, regarding the viability of the project, hence we set an IRR of 20% to help dissuade any doubts of technology risk and help us acquire the necessary level of capital.

### Setting a benchmark and formulating pricing assumptions

We will use conventional energy as a benchmark when analyzing both models’ benefits and costs. According to the Energy Information Administration (EIA) data from 2009, the average American household consumes 936 kilowatt hours of electricity per month at an average retail price of 10.65¢ per kilowatt hour. This implies that average household consumes $99.70 worth of electricity a month. However, given the specific geographic locality of California, the average household spends $139.56 in electricity a month. Helping to put this into context, the EIA states that the United States produces 4,156,745 (thousand) Megawatt hours (MWh) per year of which 48.5% comes from coal, 21.6% from natural gas, 19.4% from nuclear, 5.8% from hydroelectric sources, 1.6% from oil and 3.1% from others, such as solar and wind energy [16].

Since 1970, as sustained by Black, the retail price of residential electricity in California has risen by an average of 6.7% annually. For our analysis, we assume that this trend will continue for the next 25 years. Under this criterion we expect the price of energy to be 22 cents per kWh by 2015, 42 cents per kWh by 2025 and 80 cents per kWh by 2035. Furthermore, we assume a discount rate of 7%. This rate represents the opportunity cost of investing in a risk free asset plus an extra 2% to accommodate price shocks to electricity. Using these values we estimate the total cost of energy for our model city at a present value of $3,471,909,155. This value represents the aggregate cost of supplying electricity to our 1,044,000,000 kWh town for 23 years. Performing the same calculations for the Sterling Dish farm and taking into consideration the necessary increase in power supplied due to transmission loss, we calculate/find a net present value of $3,763,352,167. These results will be used when comparing the costs of the photovoltaic and Stirling engine models.

### Setting two discount factors

In order to properly discount for the two technologies we are going to use two separate discount factors. For the home photovoltaic system we will assume the same discount rate we used for discounting energy coming from the national grid, 7%. Here again we assume an initial 5% discount, which measures the opportunity cost of investing in a risk free asset. However, the additional 2% represent the uncertainty in the future price of raw materials such as silicon. For the Stirling engine technology we are going to use a 10% rate. The higher discount rate makes sense in this case due to the higher upfront capital costs and the fact that there is uncertainty due the scale of this endeavor because nothing of this sort has been yet implemented.

## Results and discussion

### The case for distributed photovoltaic generation

Distributed electricity generation is an attractive technology. By reducing or eliminating dependence on the national power grid, the consumer may provide for his or her own electricity demand at essentially zero marginal cost, whilst often recouping the initial capital investment associated with setting up the generation system in future electricity savings and in the value of electricity sold to the power grid.

Photovoltaic solar power is the quintessential distributed generation technology. The power produced by a photovoltaic array scales linearly with the area of the system, so as long as the array produces enough revenue to compensate for the non-generating sunk cost of the system (the inverter, etc.), a photovoltaic array is a sensible economic choice. The only trait required of a location is open, south-facing space for installation when in the northern hemisphere. They have very low maintenance costs, require little attention from their owner, and have a lifespan of 25 years, commensurate with the time horizon of many home-planning decisions –most mortgages are 15 or 30 years.

Unfortunately, commercially available photovoltaic cells remain very expensive for most residential consumers. The key to making photovoltaic arrays a cost-effective alternative to fossil fuels lies in two economic maneuvers on the part of the federal and California state governments.

First, the United States Congress has mandated that a technology and accounting practice called “net metering” be available to all electricity consumers [17]. Under a net metering scheme, any consumer attached to the power grid is given credits for electricity that user produces above his or her own electricity consumption through the use of distributed generation technology. When the consumer is using more electricity than he or she is producing, the electricity is purchased at the normal rate. Then, at the end of the billing period, the credits are subtracted from the bill, and the consumer only owes the utility the difference between the value of the electricity he or she produced and the value of the electricity he or she consumed. Due to net metering, a photovoltaic array allows a consumer to continue to consume electricity, but at a lower price than he or she would purchase that electricity from the local utility company. These savings in future electricity bills add to the value of an installed photovoltaic array.

Second, both federal and state governments provide subsidies for the installation of solar electricity generating systems. The federal government provides a 30% tax credit, for the value of installed residential and commercial photovoltaic systems [18]. This subsidy discounts the taxes of a property owner who installs a photovoltaic system by 30% of the total price of the installed system; for the purposes of our analysis, this is equivalent to the federal government paying 30% of the cost of the photovoltaic array, leaving the remaining 70% to be paid for by state subsidies and the property-owner.

In California, the cost of installing a photovoltaic system is $8.20 per watt of generating capacity, the second lowest in the nation [19]. This cost is increased by the only substantial maintenance cost associated with residential photovoltaic systems: the replacement of the inverter. The price of a solar array inverter is 71.9¢ per watt of generating capacity [20]. Assuming 2% inflation and a 7% discount rate per annum, the present value of this replacement is 38.6¢ per watt. As the price of inverters has been dropping over time, this allowance for inverter replacement will also allow for some routine inverter maintenance in addition to the inverter replacement midway through the 25-year span of this analysis. This increases the cost of each installed watt by 39¢ per watt, bringing the total cost per installed watt of photovoltaic generating capacity to $8.69.

This cost is very high when compared to the cost of grid electricity to residential consumers, at 14.9¢ per kilowatt hour [21]. Given our predetermined assumptions that the array receives five equivalent noontime hours of sun exposure on an average day and has a 25-year lifespan, the lifetime productivity of one watt of photovoltaic generating capability is 45.6 kilowatt hours. Following our other assumptions of a 7% annual discount rate and a 6.7% increase in the cost of electricity, the present value of those generated watts is $5.92; this is only 68.9% of the initial capital investment required to acquire that one watt of generating capacity. However, after the 30% federal tax credit, the array has paid for itself, leaving a 21¢ cost to the consumer per installed watt of generating capacity. This means the lump-sum rebate given by the state of California through its California Solar Initiative is almost entirely profit for the consumer, leaving the present value of an installed watt of photovoltaic generation capacity as substantially positive.

This analysis is complicated by the way California has structured its rebate. The level of the California Solar Initiative incentive drops as more solar arrays are installed in the state, and these drops are not applied uniformly across the state. The current rebate for residential consumers ranges from $1.10 to $1.90 per watt of installed generating capacity, depending on the consumer’s utility company [22]. This level of subsidy leads to a profit for the consumer of $0.90 to $1.70 per watt of installed solar generating capacity; a 10.4% to 19.7% return on investment. In the future, this rebate is scheduled to drop as low as 20¢ per watt, but even in this case the present value of each installed watt is almost exactly zero. However, by the time the California Solar Initiatives have reached this low level of subsidy, the technology’s efficiency and cost will likely have improved enough for the photovoltaic array to remain a profitable investment. See Additional Files 2 and 3 for capacity and present-value calculations.

The meaning of these numbers is more readily grasped by considering the case of a typical home. The average Californian residence consumes 580 kilowatt hours of electricity per month, or just under two-thirds the national average. By way of comparison, the average American residence consumes 936 kWh of electricity monthly [23]. At 14.9¢ per kilowatt hour, the annual electricity bill of the average Californian residence is $1037.04. In order to meet fully the annual electricity needs of such a home, it would need a photovoltaic array capable of capturing an average of 3.81 kilowatt during the approximately 5 daily noontime hours available to all Californians During these hours, each square meter of California receives at least 5 kilowatts of power from the sun. Since our constraining assumptions establish that our solar array is 13% efficient and captures no energy outside noontime sun, a photovoltaic system of 29.3m^2^ (302 ft^2^) would power the needs of the average Californian residence.

By comparison,according to ABC New’s report, the average American house is 2349 ft^2^ in area. Assuming the average house has two stories of equal size, an array covering only slightly more than one-quarter of the house’s roof will meet the needs of the average American home in California.

At an initial capital cost of $8.20 per watt, a 3.811 kilowatt system will have a total cost of $31,251. Deducting the 30% federal tax credit reduces the capital cost to $21,875. This cost is further reduced by the California Solar Initiative rebate, which reduces the cost to between $14,635 and $17,683 for the consumer. However, since an array of this size will fully meet the annual needs of the consumer (after annual net metering), the present value of 25 years of electricity bills must be considered. Given our constraining assumptions of a 6.7% annual increase in the price of electricity, a 7% discount rate, and a loss to generating capability of .9% per year, the present value of future electricity savings is $22,581. As these future savings are greater than the out-of-pocket costs to the consumer, installing such an array is a revenue-positive action on the part of the homeowner, earning him or her $4,897 to $7,946. After a single inverter replacement halfway through the 25-year lifetime of the array, this present value is reduced to $3,411 to $6,475. However, this consumer surplus came at a loss to federal and state governments of $13,567 to $16,616. This means each grid-neutral home creates a dead weight loss of $10,157 –Of course, this money does not evaporate, it goes to another agent, the photovoltaic array-producing firm. However, it is a loss to the system between consumers and the government.

Even in situations where the present value of future savings on electricity is less than zero, additional incentives remain for homeowners to purchase photovoltaic arrays. The most substantial of these is the boon to home resale value. While estimates vary on the precise level of increase in property value due to the installation of an array, the most common estimate is that decreases in annual operating cost increase home value by a ratio of 20:1. That is to say, an array that made a home grid-neutral would decrease the average California residence’s annual electricity bill by $1,037, leading to a $20,741 increase in the property’s resale value. The logic underlying this figure is that the annual savings allow the potential homeowner to take a larger mortgage to purchase the home, and the roughly $1,000 saved each year may be put into debt service on a 5% mortgage. A more theoretical analysis would conclude that the maximum increase in property value should equal the present value of remaining future electricity bills at the time of the transfer of ownership of the house. In either case, installing a photovoltaic array is revenue-positive decision for the current owner of the house even if the home is sold the day after the array is installed.

It is important to note that these estimates are somewhat conservative given our constraining assumptions that the array has no value after its 25 year lifespan, that it generates no electricity outside of noontime hours, that the array is in the parts of California that receive the least intense sunlight, and that this study does not take into account tiered electricity pricing since it is only active in some parts of California.

In most cases, tiered pricing on retail electricity will make solar technology more attractive rather than less for most residential settings; in variable cost schemes, the price of electricity tends to be highest during the heat of the day, especially in the summer. At these times, photovoltaic arrays are at their most productive, and are likely to be producing more power than the attached home is consuming. As a result, the array will be pushing electricity onto the grid, generating net-metering credit when electricity is at its highest price. After sunset, when the photovoltaic array is not generating electricity, the residence will be drawing electricity from the grid when the price level is lower.

Of course, the most compelling reason for the widespread adoption of solar electricity generation technology is the reduction of the negative externalities of other sources of electrical power. In particular, the carbon dioxide released by the burning of fossil fuels is understood to be the driving force behind global warming, and is thus a matter of prime concern. For instance, one kilowatt hour of power generation in California correlates to 0.30 kilograms (0.66 pounds) of CO_2_ emissions, meaning a grid-neutral photovoltaic array attached to the average California residence initially reduces carbon emissions by 2.1 metric tons per year. Over the 25-year lifespan of the array, accounting for decay in the quality of the land, total CO_2_ emissions are reduced by 45.6 metric tons. This equates to 12.2 kilograms of lifetime CO_2_ emissions reduced per watt of installed generation capacity. The initial capital cost of these CO_2_ emission reductions is 67¢ per kilogram over the lifetime of the array; the federal tax credit is 20¢, the California Solar Initiative rebate is 9¢ to 16¢, and the present value of consumer net revenue per kilogram of reduced CO2 emissions is 7¢ to 14¢, depending on the level of state subsidy. The economy-wide cost of these reduced emissions is thus 22¢ per kilogram.

This analysis reveals that heavy subsidies from federal and state governments have made photovoltaic arrays a sensible investment for the average residential consumer. If the consumer possesses the available roof space facing in an appropriate direction, a photovoltaic array is a profitable investment yielding 10-20% returns over the lifespan of the array, even after a 7% discount rate, and conservative estimates for the output of the array. Even as subsidies decrease, the increase to a home’s property value provide a strong incentive for homeowners to augment their homes with grid-tied photovoltaic arrays. These returns compare particularly favorably to other investments, as they are not subject to taxation; federal law mandates that photovoltaic arrays do not increase property taxes, and the present value of future electricity savings are already post-tax earnings.

### The case for concentrated dish Stirling generation

The size of our solar farm is determined by the number of Stirling engines needed to power our model city and the manner in which these will be arranged. Each dish Stirling engine produces 25kilowatts on its own [23] given that our model city requires 130 megawatts we would require 5,200 dish Stirling engines. Note that the construction of a solar farm is systematic and allows for each completely installed dish to begin generating electricity prior to the full completion of the farm (see Additional File 4). In this case we have established that the dishes will be installed in sets of 60, each one ramping to productive capacity when installed. Hence, 86 2/3 60-dish installations are required, which we will round up to 87 to cover for extra energy spikes, other engines lost due to maintenance, etc.

Taking conventional estimates into consideration we determine that the plant would required between 780 and 910 acres to accommodate the number of dishes necessary to power our farm sustainably. Note that the traditional means of calculating the dimensions required for a plant, as explained by Gallagher, is to assume 6 to 7 acres per 1MW. To add precision for the sake of later calculations, we will choose 6.5 acres as the requirement per megawatt. Given this we calculate a land requirement of 845 acres. Since our solar farm has been set just outside the city of Barstow in the San Bernardino County, where the expected cost of land is of $974 per acre (See Constraining Assumptions of Dish Stirling System for details), we estimate a cost of $823,030 in order to fully house the required equipment.

According to Sean Gallagher, a 130 megawatt plant size would roughly necessitate 150 construction workers. Due to the nature of the construction we fortunately would not need a specialty construction company or a wealth of engineers. Another bonus of this well-defined, modular construction process is that it allows for 24-hour construction as the optical alignment can take place during the night. See Additional File 4 for details on the construction process.

The construction progresses at a typical speed of one megawatt of generating capacity completely installed and completed per day. Given that each dish represents 25 kilowatts (or 0.025 MW) we get a number of 40 dishes installed per day. This allows for four arrays of 60 to go active every week. Now, assuming completion of 40 dishes a day, and given 5,200 dishes required, the construction process would stretch over 130 days.

There is some difficulty in cost speculation regarding construction as well as parts production related to dish Stirling. This uncertainty stems mostly from the lack of any large-scale plants having been put into commission. Even so we have analyzed the costs associated with similar large-scale construction projects and have come up with the following information.

Port, in a 2005 BusinessWeek article stated that the handcrafted dish itself is a costly monster at $250,000 per rig. Bulk orders, opposed to the one-off tailor made orders, can help lower the costs by roughly $100,000 apiece. Large economies of scale in production promise to lower the cost even further in theory, reaching a sticker price of roughly $80,000 or even $50,000. Further research has shown that the new expectation for “mature price approximation” for the strict production of dish Stirling engines is $1,000 per kilowatt [24], given larger scale production. This number fits well with the cost adjustments achieved with larger installations. Sean Gallagher cited the notion that a 25kilowatt dish Stirling engine costs $75,000 per dish installed – including both the fabrication and installation costs. This gives a price of $3,000 per kilowatt. This discrepancy of $2,000 can be accounted for by different production cost approximation and the cost of installation. Therefore, given the situation today we estimate a cost of $75,000 for each engine in an ideal production cycle. This implies a cost of $75,000*5,200, equaling $390,000,000 for both dish production and installation.

However, any substantial exploitation of the renewable source will depend on being able to transmit the energy from its source to its final point of usage, in this case, an urban center [25]. Hence, a substation needs to be constructed in order to lower the voltage transmitted by the solar farm. Placing the solar farm roughly one hundred miles from our city means that we need a minimum transmission voltage of 138,000 volts. For the initial calculation we are using a base unit for a 40-megawatt plant and given that these costs are linear we can then adjust for our 130-megawatt solar farm. Assuming high side protection, a circuit breaker will need to be installed which will cost $75,000. Then at the heart of the substation we have the transformer. A 138Kv to 12.5Kv 40 MVA transformer is going to cost $750,000. In addition there is a low side breaker, which recent estimates put at $20,000. Now that we have the large pieces of capital accounted for there is the engineering and parts and pieces need to connect it all together and make it work. A conservative estimate was given of $155,000, which brings our grand total to $1,000,000 for our 40 megawatt substation. Adjusting for our 130 megawatt farm leaves us with a fixed cost of $3,250,000.

As to maintenance costs, these will be calculated on a kilowatts per hour basis, which requires an estimate of the kilowatts per hour received per day. Barstow in San Bernardino County, CA enjoys an average number of 7.587 kWh/m^2^/day [26]. Knowing that each dish Stirling engine is 38 foot high by 40-foot wide solar concentrator in a dish structure [27], we calculate a surface area of about 111m^2^. Given that this system has an efficiency rating of 31.25% for converting solar thermal heat into grid quality electricity [28], we calculate that out of a total of 7.587 kWh/m^2^/day hitting Barstow only 2.37 kWh/m^2^/day will be converted into grid ready electricity. Hence, 96,058.5 kilowatt hours per year can be generated per dish.

Given our established maintenance cost of 1.8¢ per kilowatt hour, we get a maintenance cost of $1,729.1 per dish per year and a total cost of $8,991,078.67 per year for the 5,200 dishes in the plant. Another way of viewing this, which this study will later use to compare it with photovoltaic cells, is $.069 per watt per year.

Therefore the present value of the maintenance cost over the predetermined lifespan of 23 years, assuming an inflation rate of 2% and a discount rate of 10%, would be 78¢ per watt or $101,855,915 for the whole 130-megawatt plant. See Additional Files 5 and 6 for present value calculations.

From an energy standpoint it appears that the solar farm is primed for commercial success – at least as far as demand is concerned. The solar source delivers very reliable peak power when the sun is shining. This time is ideal for delivery of sunlight, as daytime is the end of the user’s peak demand: therefore, peak load equals peak power.

In order to calculate the lifetime profitability of the plant we must take into account the construction costs as well as the fixed costs and upfront capital required for the initial construction. Given the quick nature of the construction process we would need the construction cost, the substation cost, and the cost of the land upfront. In order to acquire this level of capital from investors we must appeal to them with an attractive internal rate of return based on the perception of risk associated with the technology. As stated before, this study has assumed that an IRR of 20% would help dissuade any doubts of technology risk and allow for us to acquire the necessary level of capital.

In order to derive the revenues generated by the Stirling engines technology we used the total energy needed per year for our city: 1,044,000,000 kilowatt hours. Following our constraining assumptions we used a high side estimate of 8¢ per kilowatt-hour, 6.7% increase in electricity per year and 10% discount rate, arriving at a revenue of $1,402,282,942.

Using the above calculations for capital, land and the substation, we arrived to a total fixed cost of $394,073,030. Given that all this money is borrowed upfront we are giving our investors an internal rate of return of 20%. Total interest payment to investors is $78,814,606. Finally, we must account for maintenance cost, which has a present value of $101,855,915. Adding these three numbers together we arrive at a complete lifetime cost of $574,743,551.Given that profits equal revenue minus cost, that is $1,402,282,942 less $574,743,551, we arrive at total profits of $827,539,391.

### A discussion on potential problems of solar technologies

From the above analysis it is clear that both investments are a revenue positive action. However, some concerns may remain as to its pragmatism given that some potential problems of relying on the sun as a source of energy include seasonality, cloud cover and unpredictability, as well as nightly outages.

Though it may seem obvious there is a lack of sunlight during the evening, a problem that represents an important factor when considering solar energy, the alternative trough and solar tower CSP systems can utilize a hybridization system to combat their nighttime losses. Though less efficient, they utilize natural gas to keep their turbines moving. This is not too large a concern as it utilizes equipment that would otherwise be idle. There have been proposals for the incorporation of a hybrid fossil fuel system into the Dish Stirling system, but it would suffer from lower efficiencies and lose some of its zero emissions appeal. The notion of a mixed fuel system is a disadvantage for the Stirling, as it would need to be an integral part of its design. Regarding photovoltaic cells, although during the nighttime energy would not be produced, during the day the cells should overproduce. The net metering enables the photovoltaic cell to take advantage of electricity from the national grid during times of shortages, but due to its overproduction, stay grid neutral.

Additionally, both photovoltaic and dish Stirling technologies can fall victim to the unpredictability of cloud cover and weather. However, Dish Stirling units have the unique ability to ramp up to full output within seconds. This coupled with their bigger size and ability to track the sun, as explained by Leitner, allows for average output that tracks average radiation levels very well. Still, they suffer similar disadvantages to PV given cloud cover, but they are even worse off given their inability to utilize scattered light.

**Figure 1:**
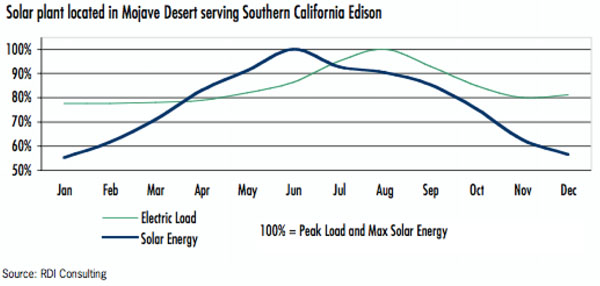
Solar resource and electric load in the Mojave Desert.

Lastly, Leitner also explains that in the case of seasonality, clouds and haze reduce output by 20% in December and January. Likewise, shorter days and less direct exposure to sunlight are instrumental in the total output of the Stirling engines. The summer remains the strongest time period for sun collection. However, despite these short falls, solar energy closely matches the electricity consumption cycle of consumers. The energy production is closely correlated with load, increasing in summer when it is most required – air conditioning being a *huge* factor in this region. The result is almost simply a downward parabola, centered at June (See Figure 1).

## Conclusions

Given our analysis in the previous sections, we conclude that the dish Stirling system is a superior option. We found that the dish Stirling consumer receives 6.37 dollars per watt while the home photovoltaic system consumer receives between 0.9 and 1.70 dollars per watt. Given these findings, we see that consumers are better off investing in a dish Stirling system. We see a significantly greater return on this technology compared to photovoltaic cells. This, at first, seems odd given that the expenses for Stirling engines are much greater than that of photovoltaic cells. However, given that the power is ultimately sold back to consumers we were eventually able to realize a profit. Furthermore, once put in the scope of the real world the dish Stirling engine appears to gain more positive moment. For example the feasibility of a solar farm, given its size, can often be brought into question. However, gaining a set of several strong investors seems much more feasible than getting a town of 150,000 households to put photovoltaic cells on their roofs. It is far easier to do the former, which intuitively makes sense. Then there is the issue of efficiencies. We said earlier that the efficiency of photovoltaic cells is between 13-16% while that of the Stirling engine is 31.25%. Based on the higher efficiency of the Stirling engine, it is not difficult to believe that this technology will outperform its rival. However, one thing we did not take into consideration was potential subsidies or grants given for the construction of the farm. These have the potential to drive the costs down even further, increasing the watts per dollar generated, thus further widening the gap between Stirling engines and photovoltaic systems.

If our goal is a reduction of CO_2_ emissions, then clearly both methods of electric productions eliminate most CO_2_ emissions via reduction of fossil fuel-based production processes. Though there may be some CO_2_ emissions during the manufacturing processes these emissions are incredibly small in comparison to the reduction in fossil fuels used.

As to policy implications, given current levels of subsidies and tax credits we found that the home photovoltaic system actually *returns* a profit to the homeowner. This indicates that these subsidies are too high and the policy is lagging behind new advances in technology. This misallocation could instead be used in the subsidy of dish Stirling farms where it would receive a much higher return.

Stepping away from subsidy policy we must now also consider the environmental impact concerns of dish Stirling construction. The clearing of vast acreages of land poses serious concerns for wildlife habitats as well as water usage issues. One must remember that these farms are located in the Mojave Desert where water is scarce. The Mojave Desert Land Trust was set up to combat the development of these precious ecosystems of the west. This group has taken the initiative to purchase land an incorporate it into preserves, saving animals from possible extinction.

Ultimately, the positive aspects seem to outweigh any minor concerns or potential externalities. The solar farm, and even the less practical decentralized photovoltaic deployments, help alleviate CO_2_ emissions as well as maturing renewable energy technology. The major goal is to one day achieve fully sustainable systems, run completely on renewable energy, giving a cheap source of electricity and an all-important source of energy independence.

## List of abbreviations used

CIBSE: Chartered Institution of Building Services Engineers; CSP: concentrated solar power; DSS: Dish Stirling Systems; EIA: Energy Information Administration; IRR: initial rate of return; kWh: kilowatt hour; mWh: megawatt hour; PV: photovoltaic cells.

## Competing interests

The authors declare that they have no competing interests.

## Authors' contributions

AH contributed with the science explanation and background of the photovoltaic technology. AJA participated in the design of the model city, performed economic analysis of fossil-based fuel that was used as a benchmark, and helped to draft the manuscript. KJP performed the economic analysis of household photovoltaic technology. JPO provided the background explanation on how Dish Stirling Systems work and contributed with the economic analysis of concentrated solar power technology. MF participated in the design of the model city and the economic analysis of fossil-based fuel. VAB compiled the data of fixed and variable costs for implementing the solar farm, contributed with the economic analysis of concentrated solar power technology, and helped draft the manuscript. All authors participated in the design of the study, performed the final analysis, read and approved the final manuscript.

## Supplementary Material

Additional File 1 Click here for file

Additional File 2 Click here for file

Additional File 3 Click here for file

Additional File 4 Click here for file

Additional File 5 Click here for file

Additional File 6 Click here for file
