# Supplementary material for: The place of solar power: an economic analysis of concentrated and distributed solar power
Source: Chem Cent J. 2012 Apr 23;6(Suppl 1):S6. doi: 10.1186/1752-153X-6-S1-S6 (PMC3332255; doi:10.1186/1752-153X-6-S1-S6)
Supplement: Additional File 1 [file 1752-153X-6-S1-S6-S1.doc]

# The Place of Solar Power: An Economic Analysis of Concentrated and Distributed Solar Power

### Additional File 1 – Understanding Photovoltaic Technology

A photovoltaic cell is composed of two thin layers of a semi conducting material, most commonly silicon, separated by a non-conducting junction. Silicon is used because most of our solar radiation photons have energies greater than the band gap of silicon. The two layers are formed by doping, the addition of a small amount of impurities to create positive and negative layers. This construction is called a p-n junction [1]. The lower p-layer contains atoms with electrons in their outer orbital that are easily lost. The upper n-layer contains atoms that lack electrons in their outer orbital and therefore readily gain them. When a photon of light above the band gap of the material hits the layer, the electrons are excited from their valence band into the conduction band, separating the positive holes from the negative electrons, creating an electrical potential between the two layers [2]. This potential provides the energy for the electric current to flow, the electrons flow through the electrical device to the upper layer as a unidirectional flow, a direct current (DC), which needs to be converted into an alternating current (AC) so that it can be entered into the grid and utilized by appliances in the home, this is done by an inverter. See Figure 2 to notice in detail the anatomy of a photovoltaic cell.

An inverter takes the DC input and runs it into a pair (or more) of power switching transistors. By rapidly turning these transistors on and off, and feeding opposite sides of a transformer, it mimics an AC current into the transformer. The transformer changes this "alternating DC" into AC at the output, which is done by producing a magnetic field, which in turn induces an electrical current of AC nature. Refer to Figure 3 to see a simplified diagram of an inverter:

The average efficiency of these cells are said to be between 13-16%. This loss in energy results from reflectance losses, thermodynamic efficiency losses and resistive electrical losses [3]. Reflective losses refer to the radiation that bounces off of the solar cell without being absorbed or altered in anyway, it is estimated that approximately 10% of the sun’s energy is lost in this way [4]. Thermodynamic losses arise where photons with energy lower than the band gap of the absorber material cannot generate a hole-electron pair, and so their energy is not converted to useful output and only generates heat if absorbed. For photons with energy above the band gap energy, only a fraction of the energy above the band gap can be converted to useful output. When a photon of greater energy is absorbed, the excess energy above the band gap is converted to kinetic energy of the carrier combination. The excess kinetic energy is converted to heat through phonon interactions as the kinetic energy of the carriers slows to equilibrium velocity and result to up to 75% of the energy loss [5].Other factors that influence the efficiency of the cell include temperature and soiling. Increase in temperatures decreases the efficiency of the cell because the band gap of the intrinsic semiconductor shrinks decreasing the voltage by a greater amount than the increase in current that results from the increased temperature and so there is a net decrease in power.

The loss due to temperature (see Figure 4) combined with losses from dust reduces the energy output by about 10% [6]. In extreme cases, particularly where the arrays are not placed at a tilt of 15° or more, dust accumulation can result in a power reduction of 10% on its own [7]. Finally, allowances have to be made for losses in the inverter (10-15%) and as a result of electrical resistance in the wires (1-3%) [8].

**References – Additional File #1**

1. Wright RT: Renewable Resources; Tenth edition; Environmental Science, 2008.
2. **Parker SP: McGraw-Hill Dictionary of Scientific and Technical Terms, 6th edition; The McGraw-Hill Companies, Inc; Chicago, 2002.**
3. Wright RT: Renewable Resources; Tenth edition; Environmental Science, 2008.
4. Chartered Institution of Building services Engineers staff: Understanding Building Integrated Photovoltaics; Chartered Institution of Building Services Engeeneers; May 1, 2000
5. Cheng CL, Sanchez Jimenez C, Meng-Chieh L: Research of BIPV optimal tilted angle, use of latitude concept for south orientated plans. ScienceDirect 2009.
6. Harwell: A Study of the feasibility of photovoltaic modules as a commercial building cladding component; Report no. S/P2/00131/REP. Energy Technology Support Unit 1993.
7. Chartered Institution of Building services Engineers staff: Understanding Building Integrated Photovoltaics; Chartered Institution of Building Services Engeeneers; May 1, 2000
8. Harwell: A Study of the feasibility of photovoltaic modules as a commercial building cladding component; Report no. S/P2/00131/REP. Energy Technology Support Unit 1993.

## Figure 2 - Anatomy of a Photovoltaic Cell


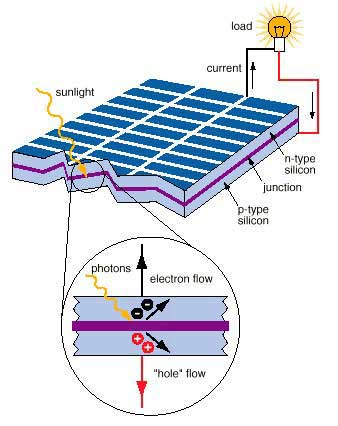


**Source:** http://zone.ni.com/devzone/cda/tut/p/id/7229

## Figure 3 - A Simple AC/DC inverter


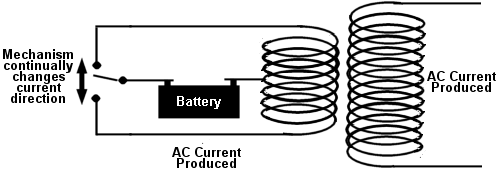


**Source:** http://www.solar-facts.com/inverters/how-inverters-work.php

## Figure 4 - Efficiency of Different Types of PV Cells as a Function of Temperature


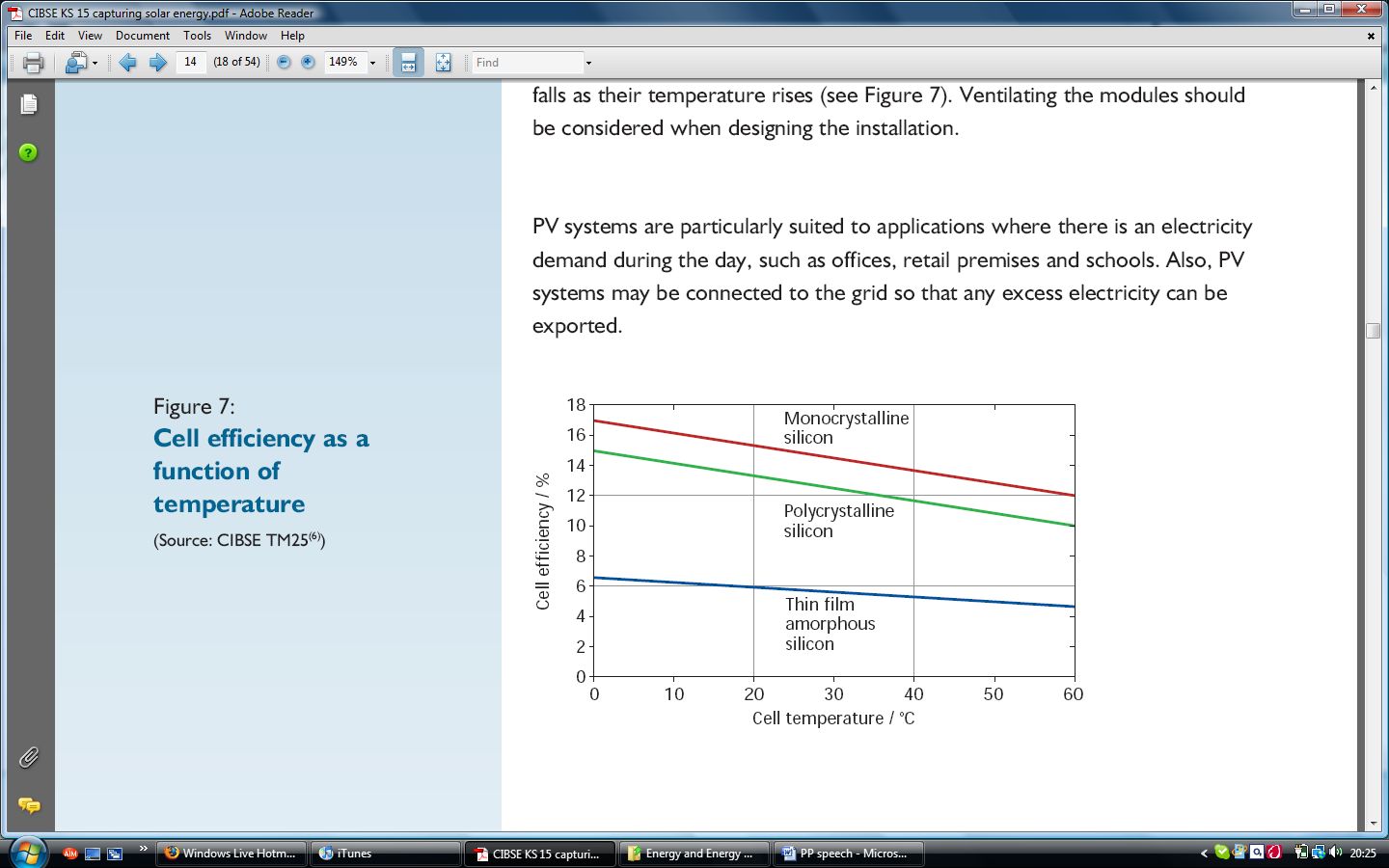


**Source:** Understanding Building Integrated Photovoltaics, CIBSE TM25: 2000
