# Supplementary material for: The place of solar power: an economic analysis of concentrated and distributed solar power
Source: Chem Cent J. 2012 Apr 23;6(Suppl 1):S6. doi: 10.1186/1752-153X-6-S1-S6 (PMC3332255; doi:10.1186/1752-153X-6-S1-S6)
Supplement: Additional File 2 [file 1752-153X-6-S1-S6-S2.doc]

# The Place of Solar Power: An Economic Analysis of Concentrated and Distributed Solar Power

**Additional File 2** – Financial Analysis of Installing a Photovoltaic Array

| Initial Cost per kWh: | 0.149 | US$ |
| --- | --- | --- |
| Home Consumption in kWh: | 580 | kWh |
| Annual consumption: | 6960 | kWh |
| Annual electricity bill: | 1,037.04 | US$ |
| Array needs to generate: | 19.05544 | kWh/day |
| Noontime solar hours per day: | 5 | Hours |
| Size of array: | 3.811088 | kW |
| Cost per installed watt: | 8.2 | US$ |
| Cost of inverter replacement per watt: | 0.39 | US$ |
| Total present cost per installed watt: | 8.59 | US$ |
| Total installation cost: | 31,250.92 | US$ |
|  |  |  |
| **Case 1: For $1.90 CSI subsidy per watt** |  |  |
| Cost after federal 30% tax credit: | 21,875.65 | US$ |
| State subsidy per watt: | 1.90 | US$ |
| Cost after $1.55/W state subsidy: | 14,634.58 | US$ |
| Plus inverter replacement per watt: | 16,120.9 | US$ |
| Annual loss in efficiency: | 0.9 | % |
| Discount rate: | 7 | % |
| Increase in electricity price: | 0.067 | US$ |
| Present value for discount rate: | 22,580.72 | US$ |
| Net Savings in Present Value: | 6,459.82 | US$ |
|  |  |  |
| **Case 2: For $1.10 CSI subsidy per watt** |  |  |
| Cost after federal 30% tax credit: | 21875.65 | US$ |
| State subsidy per watt: | 1.10 | US$ |
| Cost after $1.55/W state subsidy: | 17683.45 | US$ |
| Plus inverter replacement per watt: | 19169.77 | US$ |
| Annual loss in efficiency: | 0.9 | % |
| Discount rate: | 7 | % |
| Increase in electricity price: | 0.067 | US$ |
| Present value for discount rate: | 22,580.72 | US$ |
| Net Savings in Present Value: | 3,410.949 | US$ |
