# Supplementary material for: The place of solar power: an economic analysis of concentrated and distributed solar power
Source: Chem Cent J. 2012 Apr 23;6(Suppl 1):S6. doi: 10.1186/1752-153X-6-S1-S6 (PMC3332255; doi:10.1186/1752-153X-6-S1-S6)
Supplement: Additional File 3 [file 1752-153X-6-S1-S6-S3.doc]

# The Place of Solar Power: An Economic Analysis of Concentrated and Distributed Solar Power

**Additional File 3** - Financial Analysis of 1 Watt Installed Photovoltaic Generating Capacity

| 1 | Watt |
| --- | --- |
| 5 | Hours of noontime equivalent sun per day |
| 5 | Wh of daily electricity generation |
| 1825 | Wh per year |
| 25 | Years of generation |
| 45625 | Lifetime Wh output of 1W |
| $ 8.2 | Cost of initial installation |
| $0.39 | Cost of inverter replacement |
| $8.59 | capital cost per watt |
| $0.18827397260274 | capital cost per lifetime kWh |
| *Case 1: For a $1.10 per watt CSI rebate* | |
| Starting Price of Electricity | $0.149 |
| Rate of Change in Electricity Price | $1.067 |
| Discount Rate | 7% |
| Annual loss in efficiency | 0.9% |
| Present value of 25 years future value | $5.92 |
| Capital Cost after 30% Fed Subsidy | $5.74 |
| Value of State Subsidy/W | $1.10 |
| Cost after state subsidy | 5.03 |
| Minus present value of future savings | (0.89) |
| As a share of cost: | -10.3% |
| *Case 2: For a $1.90 per watt CSI rebate* | |
| Starting Price of Electricity | $0.149 |
| Rate of Change in Electricity Price | $1.067 |
| Discount Rate | 7% |
| Annual loss in efficiency | 0.009 |
| Present value of 25 years future value | $5.92 |
| Capital Cost after 30% Fed Subsidy | $5.74 |
| Value of State Subsidy/W | $1.90 |
| Cost after state subsidy | 4.23 |
| Minus present value of future savings | ($1.69) |
| As a share of cost: | -20% |
