# Supplementary material for: The place of solar power: an economic analysis of concentrated and distributed solar power
Source: Chem Cent J. 2012 Apr 23;6(Suppl 1):S6. doi: 10.1186/1752-153X-6-S1-S6 (PMC3332255; doi:10.1186/1752-153X-6-S1-S6)
Supplement: Additional File 4 [file 1752-153X-6-S1-S6-S4.doc]

### The Place of Solar Power: An Economic Analysis of Concentrated and Distributed Solar Power

### Additional File 4 – Understanding Dish Stirling Engines

The Dish Stirling System consists of a parabolic solar concentrator, a tracking system, a solar receiver and an engine with a generator. The parabolic concentrator reflects the incoming solar radiation onto a cavity receiver, which is located at the concentrator’s focal point and transmits it to the heat engine (See Figure 5). The engine is a sealed system filled with hydrogen or helium (a transfer medium), and as the gas heats and cools, its pressure rises and falls. The change in pressure drives the pistons inside the engine, producing mechanical power [1]. The mechanical power in turn drives a generator directly connected to the engine and converts the mechanical energy into electricity (AC) [2].

The tracking system enables the solar concentrator to follow the sun, keeping the reflected radiation at the focal point. It rotates about two axes. The orientation towards the sun is either determined by a sun-tracking sensor, or by a special computer program which predicts the position of the sun. This coupled with the dish’s ability to ramp to the grid within a minute allows it to capture the highest amount of solar energy possible [3].

Each curved glass dish will direct its reflected energy to a 25-kilowatt power generator. The Stirling dish “heat antenna” is the device of choice, as the better-known solar cells still suffer from high costs and very limited efficiency. The typical photovoltaic solar cell harvests only between 10 and 15 percent of the available solar energy, where as the Stirling-brand dish converts 29.4% [4] Further benefits include its high engine-operating temperature, which allows for air cooling, meaning there is no need for water-cooling and the associated water system required [5].

The dish Stirling hardware itself is appealing because it is the lowest cost solar electricity source available. It also provides high-value power at the time of the day of peak demand. The engine technology itself is tried and tested and the few small test beds that have been built are proving reliable and promise profitability too. Many developers within California now understand the possibilities and several solar projects are now underway, though most are still in the planning stages. One of the most ambitious is the Stirling Energy Systems plan for California’s Mojave Desert. The upstart from Phoenix plans to build the world’s largest solar farm. Southern California Edison, the largest buyer of renewable energy in the West, has committed to a 20-year contract, stipulating the sale of all the electricity the 500 megawatt facility can produce.

The construction itself does not necessitate specially trained workers/engineers. This is because the process involves a very systematic installation process. First the pedestals are installed – vibrated into the ground via low frequency vibrations. Next the workers establish the electrical system and hook them to the farm’s system. The following step involves a fuel system, which provides liquid to the pedestal allowing the engine to operate, and the final step is an assembly line for erecting the solar dishes themselves. This process allows for 24-hour construction as the optical alignment can take place during the night.

1. Sandia National Laboratories: Sandia, Stirling to build solar dish engine power plant; New Center, 2004. [<http://www.sandia.gov/news-center/news-releases/2004/renew-energy-batt/Stirling.html>]
2. Schlaich Bergermannund and Partners Structural Consulting Engineers: EuroDish – Stirling System Description; 2001.
3. Schlaich Bergermannund and Partners Structural Consulting Engineers: EuroDish – Stirling System Description; 2001.
4. Port O: Solar Power’s New Hot Spot; *BusinessWeek*; August 19, 2005. [<http://www.businessweek.com/technology/content/aug2005/tc20050819_0041_tc024.htm>]
5. Leitner A: Fuel from the Sky: Solar Power’s Potential for Western Energy Supply; National Renewable Energy Laboratory; 2002.

## Figure 5 - Simple Diagram of a Dish Stirling Engine


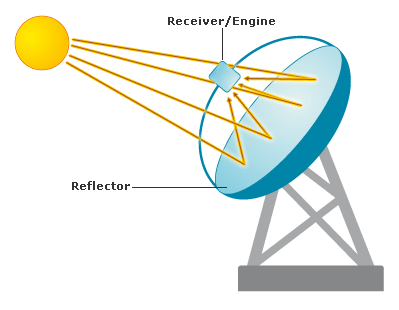


**Source:** http://www.abengoasolar.com/sites/solar/en/abengoa_solar_nt/
