# Supplementary material for: The place of solar power: an economic analysis of concentrated and distributed solar power
Source: Chem Cent J. 2012 Apr 23;6(Suppl 1):S6. doi: 10.1186/1752-153X-6-S1-S6 (PMC3332255; doi:10.1186/1752-153X-6-S1-S6)
Supplement: Additional File 5 [file 1752-153X-6-S1-S6-S5.doc]

# The Place of Solar Power: An Economic Analysis of Concentrated and Distributed Solar Power

**Additional File 5:** Calculating the Present Value of Dish Stirling Engine Maintenance in Cents per Watt Capacity (23 years)

| **Inflation rate^ year** | **Discount rate** | **year** | **Discount rate ^ year** | **I. rate^year / D.rate^year** | **Present Value (cents/watt)** |  | **Inflation level:** | 2% |
| --- | --- | --- | --- | --- | --- | --- | --- | --- |
| 1.000 | 1.1 | 0 | 1.000 | 1.000 | 0.069 |  | **Discount Rate:** | 10% |
| 1.020 | 1.1 | 1 | 1.100 | 0.927 | 0.064 |  | **Price ($)** | 0.069 |
| 1.040 | 1.1 | 2 | 1.210 | 0.860 | 0.059 |  |  |  |
| 1.061 | 1.1 | 3 | 1.331 | 0.797 | 0.055 |  |  |  |
| 1.082 | 1.1 | 4 | 1.464 | 0.739 | 0.051 |  |  |  |
| 1.104 | 1.1 | 5 | 1.611 | 0.686 | 0.047 |  |  |  |
| 1.126 | 1.1 | 6 | 1.772 | 0.636 | 0.044 |  |  |  |
| 1.149 | 1.1 | 7 | 1.949 | 0.589 | 0.041 |  |  |  |
| 1.172 | 1.1 | 8 | 2.144 | 0.547 | 0.038 |  |  |  |
| 1.195 | 1.1 | 9 | 2.358 | 0.507 | 0.035 |  |  |  |
| 1.219 | 1.1 | 10 | 2.594 | 0.470 | 0.032 |  |  |  |
| 1.243 | 1.1 | 11 | 2.853 | 0.436 | 0.030 |  |  |  |
| 1.268 | 1.1 | 12 | 3.138 | 0.404 | 0.028 |  |  |  |
| 1.294 | 1.1 | 13 | 3.452 | 0.375 | 0.026 |  |  |  |
| 1.319 | 1.1 | 14 | 3.797 | 0.347 | 0.024 |  |  |  |
| 1.346 | 1.1 | 15 | 4.177 | 0.322 | 0.022 |  |  |  |
| 1.373 | 1.1 | 16 | 4.595 | 0.299 | 0.021 |  |  |  |
| 1.400 | 1.1 | 17 | 5.054 | 0.277 | 0.019 |  |  |  |
| 1.428 | 1.1 | 18 | 5.560 | 0.257 | 0.018 |  |  |  |
| 1.457 | 1.1 | 19 | 6.116 | 0.238 | 0.016 |  |  |  |
| 1.486 | 1.1 | 20 | 6.727 | 0.221 | 0.015 |  |  |  |
| 1.516 | 1.1 | 21 | 7.400 | 0.205 | 0.014 |  |  |  |
| 1.546 | 1.1 | 22 | 8.140 | 0.190 | 0.013 |  |  |  |
|  |  |  |  |  | **0.782** |  |  |  |
