# Supplementary material for: The place of solar power: an economic analysis of concentrated and distributed solar power
Source: Chem Cent J. 2012 Apr 23;6(Suppl 1):S6. doi: 10.1186/1752-153X-6-S1-S6 (PMC3332255; doi:10.1186/1752-153X-6-S1-S6)
Supplement: Additional File 6 [file 1752-153X-6-S1-S6-S6.doc]

### The Place of Solar Power: An Economic Analysis of Concentrated and Distributed Solar Power

### Additional File 6: Stirling Engine Top-Level Cost-Benefit Calculations in Present Value (23 Year Lifespan)

| Solar Farm Aggregate Revenue | $1402282942 |
| --- | --- |
| Aggregate Maintenance Cost | $101855915 |
| Substation | $3250000 |
| Capital | $390000000 |
| Land | $823030 |
| Total | $394073030 |
| IRR for investors | $78814606 |
| Maintenance | $101855915 |
| Complete life time costs | $574743551 |
|  |  |
| Profits | **$827539391** |
